# Vitamin Status and Risk of Age-Related Diseases Among Adult Residents of the Pearl River Delta Region

**DOI:** 10.3390/nu17101637

**Published:** 2025-05-10

**Authors:** Yongze Zhao, Siqian Zheng, Bohan Wang, Wenhui Xiao, Ping He, Ying Bian

**Affiliations:** 1 Department of Public Health and Medicinal Administration, Faculty of Health Sciences, University of Macau, Taipa, Macau 999078, China; 2 Institute of Chinese Medical Sciences, University of Macau, Taipa, Macau 999078, China; 3 State Key Laboratory of Quality Research in Chinese Medicine, University of Macau, Taipa, Macau 999078, China; 4 Unit of Psychiatry, Department of Public Health and Medicinal Administration, Institute of Translational Medicine, Faculty of Health Sciences, University of Macau, Taipa, Macau 999078, China; 5 China Center for Health Development Studies, Peking University, 38 Xue Yuan Road, Haidian District, Beijing 100191, China

**Keywords:** aging, nutrition, cross-sectional survey

## Abstract

**Background**: The Pearl River Delta (PRD) region in Guangdong, China, is urbanized and economically significant. Rapid development has shaped diverse dietary habits. In this densely populated area, there is an urgent need to assess vitamin status and its impact on age-related diseases. **Methods**: A total of 2646 participants (age: 50.92 ± 9.30 years; male: 64.06%) were recruited from the Pearl River Delta (PRD) region. Participants were included from 1 December 2020 to 30 November 2021. Three restricted cubic spline logistic models, interaction terms, and mediated effects analyses were used to assess the association between vitamin A, B, E, B_1_, B_2_, B_3_, B_5_, B_6_, and B_9_ between five age-related diseases: cerebrovascular disease (CVD), coronary heart disease (CHD), hypertension (HTN), dyslipidemia (DYS), and type 2 diabetes mellitus (T2DM). **Results**: Blood concentrations of nine vitamins showed a right-skewed distribution. Significant correlations were found between vitamin levels and age-related diseases across nine groups (*p* < 0.05). A J-shaped relationship was observed between vitamin levels and the risk of age-related diseases, except for the Vitamin A-HTN/T2DM, which showed Maximum Effective Concentration (MEC). Specific thresholds included: Vitamin A: 1080 ng/mL (DYS); Vitamin B_1_: 77 ng/mL (CVD), 75.5 ng/mL (HTN); Vitamin B_5_: 900 ng/mL (CVD), 600 ng/mL (HTN), 690 ng/mL (DYS); Vitamin B_6_: 82 ng/mL (CVD). The protective effect of vitamins against age-related diseases decreased with age, and higher levels of vitamins A and B_1_ correlated with increased hypertension risk in older adults (P_interaction_ < 0.01). Low Body Resilience Index (BRI) and physical activity mediated the protective effects of vitamins A and B_5_ on HTN and DYS, while no mediating effects were found for smoking and alcohol consumption. **Conclusions**: The effectiveness of multivitamin supplementation in preventing cardiovascular, cerebrovascular, and metabolic diseases may be limited in healthy aging populations. Health professionals should consider patients’ physiological conditions and blood vitamin levels to avoid overdose. More interventional studies are needed to establish causal relationships.

## 1. Introduction

Age-related diseases pose a significant health burden, especially as the population ages, becoming increasingly serious. Globally, age-related diseases are leading causes of death and disability in those aged 65 and older [1]. The 2021 Global Burden of Disease (GBD) found that age-related diseases accounted for 60% of the global disease burden [2]. The economic burden of age-related diseases is also significant, with estimates suggesting it will reach $47 trillion globally by 2030 [3]. In China, rapid urbanization and demographic aging have intensified this issue, with conditions like cerebrovascular disease, hypertension, and diabetes making a large contribution to the impairment of the comprehensive index of quality of life, particularly in economically dynamic regions such as the Pearl River Delta (PRD) [4]. While environmental and genetic factors have been extensively studied, the role of micronutrient deficiencies—especially vitamins—remains underexplored in the context of aging populations.

Vitamins are essential modulators of cellular metabolism, immune function, and oxidative balance. For example, vitamin A regulates lipid metabolism through retinoic acid signaling; however, excessive levels may paradoxically disrupt this process, increasing the risk of dyslipidemia [5]. B vitamins, such as B_5_ (pantothenic acid), act as precursors to coenzyme A, which is crucial for energy production and vascular health. Nevertheless, supra-physiological concentrations of these vitamins are a risk factor for all-cause mortality among Chinese patients with hypertension, especially among older adults and those with adequate folate levels [6]. Similarly, vitamin D deficiency is associated with increased inflammation and cardiovascular dysfunction, while vitamin E’s antioxidant properties help mitigate lipid peroxidation in cell membranes [7,8]. Despite their well-established roles, most studies focus on individual vitamins or Western populations, leaving the interactions of multivitamins and region-specific patterns in China poorly understood.

The Pearl River Delta (PRD) region, situated in the south-central part of Guangdong Province, China, is a highly urbanized and economically significant area renowned for its rapid economic development and dense population [9]. Due to the increasing aging population and diverse dietary habits in the region, residents may encounter unique nutritional challenges. Its residents exhibit dietary habits shaped by economic growth, including increased consumption of processed foods and reduced intake of fresh produce—a shift associated with micronutrient inadequacies [10,11]. China Nutrition Survey and Research revealed that Low vitamin D status is a common public health problem in mainland China, likely due to limited sun exposure and dietary preferences [12]. However, comprehensive data on multivitamin status and its association with age-related diseases in this region remain scarce. Existing studies often rely on self-reported dietary assessments, which lack the precision of biomarker-based analyses [13].

This study addresses existing gaps by investigating the dose–response relationships between blood concentrations of vitamins A, B-group, D, and E, and five age-related diseases: cerebrovascular disease, hypertension, dyslipidemia, coronary heart disease, and type 2 diabetes, among 2646 adults in the Pearl River Delta (PRD). Utilizing dried blood spot (DBS) assays validated against serum samples, we aim to: (1) define region-specific vitamin deficiency thresholds, (2) identify nonlinear associations between vitamin levels and disease risk, and (3) explore how age and lifestyle factors mediate these relationships. Our findings provide actionable insights for tailored nutritional interventions in rapidly aging populations, bridging a critical gap between public health research and clinical practice.

## 2. Materials

### 2.1. Study Design and Participants

This cross-sectional study investigates the nutritional status of multivitamins and age-related diseases within the adult population of the Pearl River Delta (PRD) region. The research is conducted through four components: questionnaires, case materials, physical examinations, and laboratory tests. Study variables were measured at every available time point for each participant from 1 December 2020 to 31 November 2021. Data analysis for the current study was performed from 1 October 2024 to 15 January 2025.

This study used a cluster random sampling method to select participants from the PRD region. The approach involved dividing the area into groups based on administrative divisions, from which communities were randomly selected (Excluding Hong Kong and Macao Special Administrative Regions). Within each selected community, households were randomly chosen, and eligible residents aged 20 to 88 years with a body mass index (BMI) of less than 40 kg/m^2^ were invited to participate. This methodology ensured a representative sample of the PRD population, facilitating an accurate assessment of vitamin status and its association with age-related diseases. The questionnaires were adapted from the China Public Health Service Program—Stroke Screening and Intervention for High-Risk Populations, a nationally standardized protocol with validated components for demographic, lifestyle, and medical history assessment. All staff responsible for administering the questionnaires received specialized training before the commencement of the survey.

Case Materials refers to diagnostic or hospitalization records that were provided by provincial, prefectural, and community or rural health facilities, which were used to verify disease status, with a material provision rate exceeding 85%.

Physical examinations and biospecimen collections were conducted by qualified medical professionals, including doctors and nurses who had received appropriate medical training. These examinations included assessments of general physical signs (Participants’ height and weight were measured with a weighing scale and tape measure), on-site blood pressure measurements (with at least two readings taken from the same arm), cardiac auscultation, and electrocardiograms. Further examinations were performed if murmurs were detected during cardiac auscultation or if abnormalities, such as irregular heart rhythms, were identified.

**Figure 1:**
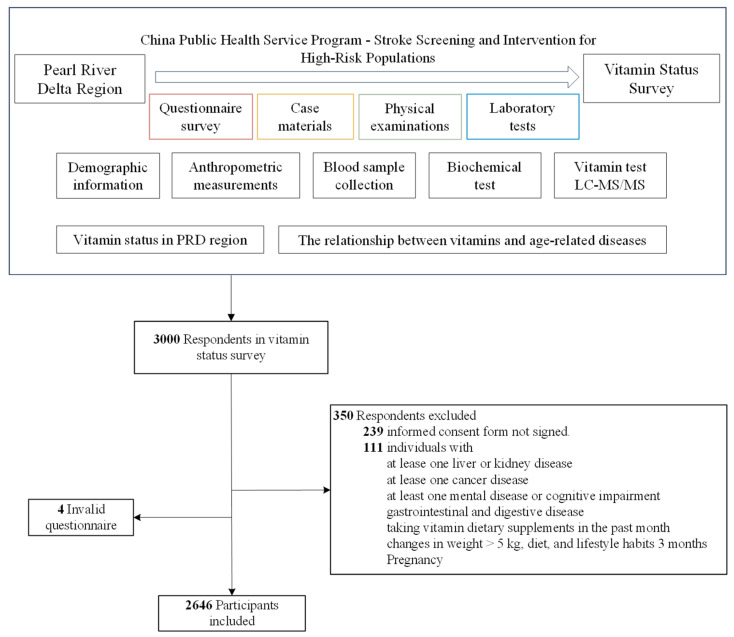
The flowchart of this study design.

The analysis of laboratory samples was entrusted to professional technicians from the Department of Laboratory Medicine at Zhuhai People’s Hospital, a tertiary-level facility. The survey results for each participant were entered separately by two professionals using Epi-Data version 3.1 software, with a data manager responsible for overall data verification. These double-check measures were implemented to minimize bias in the results. In the present study, we included 2646 participants for analysis. Figure 1 shows a comprehensive overview of the sample processing flow.

To ensure the privacy and confidentiality of participants, each respondent in the survey was assigned a unique anonymized identification (ID) number. This anonymized ID replaced the dataset’s personally identifiable information (PII). All methods concerning human participants in our study were conducted following the ethical standards laid out in the 1964 Declaration of Helsinki and its subsequent amendments. Ethical approval for this study was obtained from the Institutional Review Board (IRB) at Zhuhai People’s Hospital. The IRB approval number is 2020-8, approved on 2 November 2020. This study is registered in the Chinese Clinical Trial Registry (ChiCTR2100047303) and approved on 12 June 2021. Written and signed informed consent was obtained at recruitment from all participants, including legal representatives for illiterate participants. The study followed the Strengthening the Reporting of Observational Studies in Epidemiology (STROBE) guideline for cross-sectional studies (Additional File S1).

### 2.2. Blood Biochemistry Test

Laboratory tests include routine blood biochemical tests and blood vitamin level tests. The routine blood biochemical tests include fasting blood glucose, glycosylated hemoglobin (HbA1c), lipid profile, and homocysteine (Cys) levels. The lipid profile consists of triglycerides, total cholesterol, low-density lipoprotein cholesterol (LDL-C), and high-density lipoprotein cholesterol (HDL-C). All routine blood biochemical tests were performed in strict accordance with the relevant technical specifications.

Blood vitamin tests included fat-soluble vitamins (A, D, and E) and water-soluble vitamins (B_1_, B_2_, B_3_, B_5_, B_6_, and B_9_). Each participant had a whole blood sample collected from a fingertip to create a dried blood spot card; the type of sample tested was whole blood. All collected dried blood spot (DBS) samples were tested within 6 h using a dry blood spot (DBS)-based liquid chromatography–mass spectrometry (LC-MS/MS) method (BYHEALTH Co., Ltd., Guangzhou, China). The testing instrument was accredited by ISO/IEC 17025 [14], which outlines the requirements for the Competence of Testing and Calibration Laboratories, China National Accreditation Service for Conformity Assessment (CNAS).

There are generally well-defined standards for deficiencies or insufficiencies of fat-soluble vitamins, and these concentrations can be detected through blood assessments. China has released the methods for Vitamin A (WS/T 553-2017 [15]) and Vitamin D Deficiency in Population (WS/T 677-2020 [16]). The definitions of fat-soluble vitamin deficiency or insufficiency are typically based on serum samples. Therefore, the vitamin levels in serum and whole blood samples from 150 individuals were simultaneously tested in the preliminary phase of this study. The methodological validation of LC-MS/MS adhered to the European Medicines Agency (EMA) guidelines for validating analytical methods. The values for fat-soluble vitamins defined for whole blood, converted according to serum determination criteria, are as follows: vitamin A < 297.8 ng/mL, vitamin D < 16.5 ng/mL (male) or <17.7 ng/mL (female), and vitamin E < 3.42 μg/mL. Detailed measurements of vitamins A, B, D, and E are provided in Additional File S2. In contrast, the definitions of deficiency or insufficiency for water-soluble vitamins remain controversial and challenging within the research community. Assessing these deficiencies may require a combination of factors, including dietary history, biochemical markers, and clinical symptoms.

### 2.3. Definition of Age-Related Diseases

According to the Global Burden of Diseases, Injuries, and Risk Factors Study (GBD), from 1990 to 2021, the burden of chronic diseases in China has shown a significant upward trend. In 2021, approximately 10.64 million people (95% confidence interval: 9.05 to 12.22 million) are projected to die from chronic diseases in China, accounting for 91.0% (90.4% to 91.7%) of total deaths. Among the major causes of death from chronic diseases in China in 2021, cardiovascular diseases, cancer, and chronic respiratory diseases accounted for 79.4% of deaths, while hypertension contributed to 25.2% and diabetes to 9.7% [2]. Based on this evidence, we have selected five age-related diseases: cerebrovascular disease (CVD), coronary heart disease (CHD), hypertension (HTN), dyslipidemia (DYS), and type 2 diabetes mellitus (T2DM) for further study. CVD included cerebral infarction, cerebral hemorrhage, subarachnoid hemorrhage, and transient ischemic attack. CHD included coronary heart disease with or without atrial fibrillation (AF), and heart valve disease. The disease data were not self-reported; they were derived from medical records that included physician-confirmed diagnoses at the time of the survey. Additionally, congenital factors were excluded from the disease characteristics of the studied population.

### 2.4. Covariates

Covariates included sex (female or male), age (years, continuous), education (Primary School and Below/Middle School/High School/Undergraduate/Postgraduate and Above), smoking (yes or no), alcohol consumption (yes or no), exercise (yes or no), family history of disease (yes or no, corresponds to 5 diseases) and biochemical indicators. More details about these variables and the questionnaire are provided in Additional File S3.

### 2.5. Statistical Analysis

STATA version 17.0 (StataCorp LP, College Station, TX, USA) and R version 4.3.3 (R Core Team, 2024 [17]). A two-sided *p* < 0.05 was set to define statistical significance.

Descriptive statistics were initially presented to summarize the characteristics of the study participants. The results are presented as mean  ±  standard deviation (SD) for normally and non-normally distributed data. The nominal data presented are the reported number of participants along with the corresponding percentages. *p*-values for differences in characteristics among five age-related diseases (CVD, CHD, HTN, DYS, and T2DM) were assessed using ANOVA for normally distributed data, the Kruskal–Wallis test for routine blood biochemical data, and the χ^2^ test for nominal data. The Kolmogorov–Smirnov test was used to assess the normal distribution of blood vitamin concentrations (A, D, E, B_1_, B_2_, B_3_, B_5_, B_6_, and B_9_).

We used logistic regression analysis to investigate the cross-sectional associations between blood concentrations of nine vitamins and five age-related diseases. The fat-soluble vitamins (A, D, and E) were categorized into vitamin-deficient and normal groups based on their respective criteria, while the water-soluble vitamins (B-group) were divided into four distinct groups (Q1, Q2, Q3, and Q4) according to interquartile spacing. First, three different models were developed for the 45 groups described above, considering the potential impact of physical condition, lifestyle, and family history of disease on outcomes. Model 1 was a crude model. Model 2 was adjusted for sex, age, and education status. Model 3 additionally adjusted for BRI, smoking, alcohol consumption, exercise, and diseases that correspond to the history of illness and routine blood biochemical indicators. Next, groups for which all of the above models were statistically significant were analyzed for dose–response relationships using a three-time Restricted Cubic Spline (RCS) approach. The outcome metric used an odds ratio (OR) value, which indicates a potential deleterious effect when the OR is greater than 1. RCS is based on spline interpolation and polynomial regression techniques and is widely utilized in data analysis to explore nonlinear relationships among continuous variables, as well as to address interaction and threshold effects of independent variables. This method adapts to the complex patterns of the data by allowing the spline curve to bend at specific points. We set three nodes (P_25_, P_50_, and P_75_) based on quartiles and calculated the corresponding concentration thresholds. The Maximum Effective Concentration (MEC) was determined as the concentration value corresponding to the upper limit of the 95% confidence interval (CI) when the OR equals 1. Additionally, we calculated the concentration thresholds by replacing nodes (P_30_, P_60_, and P_90_) and increasing the number of nodes (P_20_, P_40_, P_60_, and P_80_). We conducted sensitivity analyses to ensure the accuracy of the threshold results.

We selected models that were statistically significant across all three logistic regression models for analysis of interaction and mediation effects. Binary Logit regression analyzed the interaction effect of age across various vitamin-age-related disease groups. When the effect of blood vitamin concentration on disease varied with different age values, an interaction effect between blood vitamin concentration and age was observed. This interaction effect was analyzed using a fully adjusted model (Model 3). Visreg (R package) was used to create visual representations of the effect of age on the different groups. The Karlsson-Holm-Breen (KHB) method was applied to assess whether body mass index (BMI), smoking, alcohol consumption, and physical activity mediated the effects among the various vitamin-age-related groups. Unlike the Sobel method, the KHB method is more appropriate for nonlinear regression analyses. BRI and vitamin concentration were treated as continuous variables, while smoking, alcohol consumption, and physical activity were considered dichotomous variables. The data included in the analysis does not contain any missing values; therefore, imputation is unnecessary.

## 3. Results

The main analysis included 2646 participants, 951 (35.94%) females and 1695 (64.06%) males. The mean (SD) age was 50.92 (9.30) years. A total of 2590 individuals (97.88%) had a Body Risk Index (BRI) value of less than 3.41. Additionally, the participants were categorized into four groups—underweight, normal weight, overweight, and obese—according to the Guidelines for the Prevention and Control of Overweight and Obesity in Chinese Adults. Among these participants, 1316 (49.74%) were classified as normal weight, 1077 (40.70%) as overweight, 190 (7.18%) as obese, and 63 (2.38%) as underweight. We observed significant differences in age among participants with cerebrovascular disease (CVD), coronary heart disease (CHD), hypertension (HTN), dyslipidemia (DYS), and type 2 diabetes mellitus (T2DM). Fifty-six participants (2.12%) had CVD, 40 (1.51%) had CHD, 540 (20.41%) had HTN, 417 (15.76%) had DYS, and 170 (6.42%) had T2DM. Descriptive statistics for total participants are presented in Table 1.

There were 502 (18.97%), 260 (9.83%), and 579 (21.88%) participants in the vitamin A, D, and E deficiency groups, respectively. The deficiencies of vitamins A, D, and E exhibited significant differences between genders. Specifically, deficiencies in vitamins A and D were significantly more prevalent in females than males, whereas vitamin E deficiencies were substantially more common in males than females. Additionally, vitamins A, D, E, B_1_, B_2_, B_5_, B_6_, and B_9_ demonstrated significant differences among age subgroups. An analysis of the nine vitamins using the Kolmogorov–Smirnov test indicated that the blood concentrations of all nine vitamins did not conform to a normal distribution (*p* < 0.001). Further details regarding the distribution of vitamins among participants are provided in Additional File S4.

**Figure 2:**
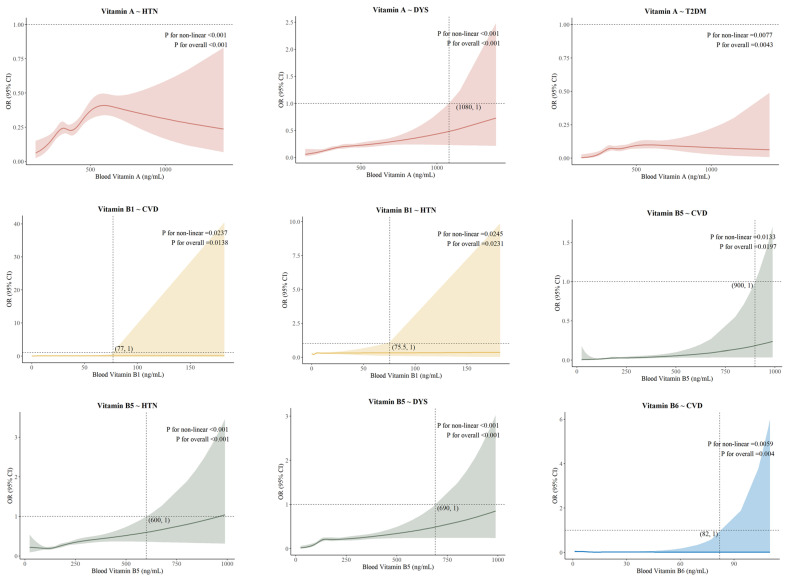
Dose–response association between vitamins and age-related diseases.

Binary logistic regression was used to investigate the relationship between vitamins and age-related diseases. In the fat-soluble vitamin group, significant differences were observed between vitamin A and HTN, DYS, and T2DM after conducting crude modeling, demographic-adjusted modeling, and further adjustments for family history of disease, lifestyle habits, and biochemical assays. In the water-soluble vitamin group (quartile grouping), significant differences were identified for Vitamin B_1_-CVD and HTN, Vitamin B_5_-CVD, HTN, DYS, and Vitamin B_6_-CVD groups in three models (Additional File S5). We examined the relationship between blood vitamin concentrations and the risk of age-related diseases using natural cubic splines for the nine groups mentioned above, revealing an overall J-shaped dose–response relationship in Figure 2. Maximum effective concentration thresholds were present in all groups except for the Vitamin A-HTN/T2DM group, Vitamin A: 1080 ng/mL (DYS; Vitamin B1: 77 ng/mL (CVD), 75.5 ng/mL (HTN); Vitamin B5: 900 ng/mL (CVD), 600 ng/mL (HTN) and 690 ng/mL (DYS); Vitamin B6: 82 mg/mL (CVD).

**Figure 3:**
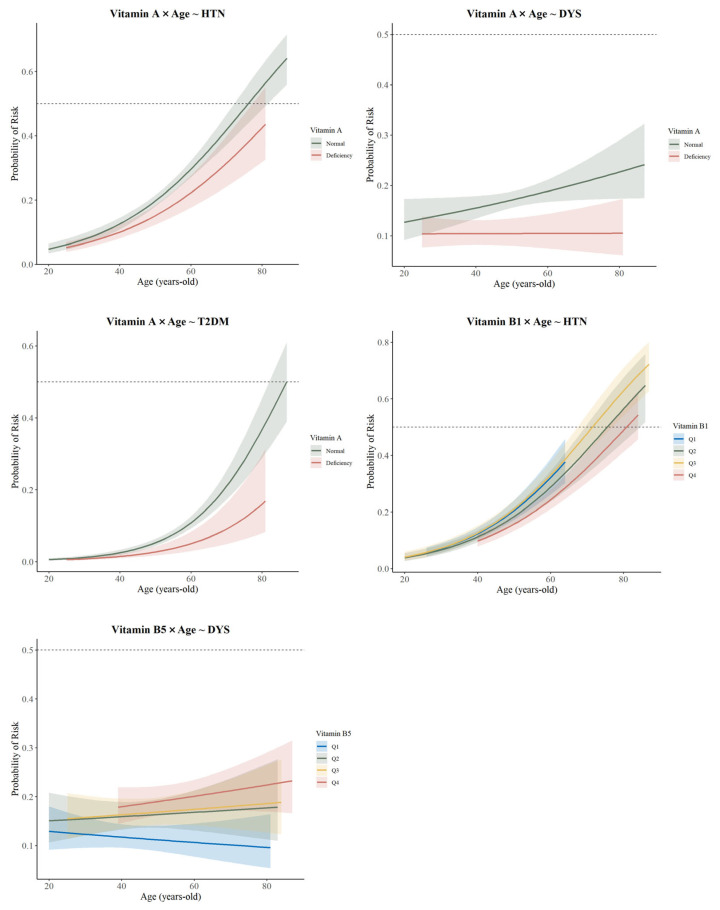
Age as an interaction term to analyze the association between vitamins and age-related diseases.

Using age as an interaction term in a fully adjusted logit model, significant differences were observed between Vitamin A-HTN (*p* = 0.036), DYS (*p* < 0.001), T2DM (*p* = 0.004), Vitamin B_1_-HTN (Q4: *p* = 0.003), Vitamin B_5_-DYS (Q2: *p* = 0.01; Q3: *p* = 0.005; Q4: *p* = 0.001) (see Additional File S6). Figure 3 indicates that the protective effect of the higher blood vitamin concentration group was less pronounced than that of the lower concentration group, and the protective effect of vitamins against age-related diseases weakened with advancing age. The results of the analyses using BRI, smoking, alcohol consumption, and physical activity as mediating variables are presented in Table 2. The groups exhibiting partially positive mediating effects of BRI included Vitamin A-HTN and DYS, as well as Vitamin B_5_-HTN and DYS. Additionally, physical activity was found to reduce the risk of CVD to some extent in DYS at high concentrations of Vitamin B_5_ and B_6_. BRI and physical activity played distinct mediating roles between blood vitamin concentrations and age-related diseases. The positive mediating effect of BRI suggested an increased risk of disease, while the negative mediating effect of physical activity indicated a reduction in disease risk.

We attempted to modify the quartiles of RCS and increase the number of nodes, but the results remained consistent with the main findings of the study. The Receiver Operating Characteristic (ROC) curve was used to evaluate the performance of the fully adjusted logistic model based on the RCS method. Except for the Vitamin A-DYS (AUC = 0.6802) and Vitamin B5-DYS (AUC = 0.6850) groups, the Area Under the Curve (AUC) values for the other groups are all greater than 0.7. The results mentioned above are presented in Additional File S7 and indicate that our findings are robust and reliable.

## 4. Discussion

### 4.1. Main Findings

#### 4.1.1. Blood Vitamin Status

The results of this study align with the national vitamin content distribution in the Chinese Residents’ Nutrition and Chronic Disease Status Report (2020) [18]. Our vitamin level survey revealed that deficiencies in vitamins A and D were significantly higher in women than in men. A GBD study supports this result, indicating that it can be attributed to the fact that women require more vitamin A for reproduction and lactation [19]. Additionally, women may be more inclined to adopt low-fat, low-calorie diets, which often lack foods rich in vitamin A [19]. A study from China found that 89% of women were vitamin D deficient [20], likely due to lower sun exposure and the impact of estrogen-containing contraceptives on vitamin D absorption [21]. Conversely, vitamin E deficiency is more common in men, possibly due to their higher animal protein intake, while women consume more vitamin E-rich foods [22]. Numerous studies confirmed that vitamin status varies by age, with the underlying reason being the body’s diminished ability to absorb and utilize vitamins as age increases [20,23,24,25]. The observed sex differences may reflect physiological demands or dietary patterns, though further studies with dietary assessments are needed to confirm these mechanisms.

#### 4.1.2. Dose–Response Associations

Except for vitamin A-HTN/T2DM, there were J-shaped associations existing between vitamin concentration and age-related disease risk, with some differences among groups. In the vitamin A-DYS, vitamin B5-CVD, vitamin B5-HTN, and vitamin B5-DYS groups, higher blood vitamin concentrations correlated with decreased protective effects. Some studies have investigated the protective effects of vitamins A and B against various age-related diseases; however, none have yielded results similar to ours [26,27,28]. Vitamin A has antioxidant activity, can scavenge free radicals, and synergize with other nutrients to regulate lipid metabolism [29]. Since vitamin A is a fat-soluble vitamin, excessive intake can accumulate in the body and interfere with lipid metabolism, which may increase the risk of DYS [30]. Vitamin B5 is essential for coenzyme A synthesis, which influences metabolic health and vascular protection [31]. Excessive vitamin B5 accumulation may disrupt physiological regulation, increase oxidative stress, and trigger inflammation, weakening its protective effects [31].

In the vitamin B1-CVD, vitamin B1-HTN, and vitamin B6-CVD groups, dose–response curves were mostly horizontal. The lack of a clear association between increased vitamin B1 levels and protection against CVD and HTN may stem from its water-soluble nature, which limits storage and necessitates continuous dietary intake [32,33]. Thus, excess vitamin B1 is typically excreted, potentially restricting its protective effects. The association between vitamin B6 levels and cerebrovascular disease risk is complex; both low and high levels can be harmful. Low levels increase the risk of CVD, while high levels may not offer additional benefits and could cause other health issues [34].

In the vitamin A-HTN/T2DM groups, we found that as blood vitamin A concentrations rose, the OR for HTN and T2DM initially increased and then decreased, while the vitamin A-HTN dose–response curve was more pronounced. This contrasts with some studies, as most indicate a U-shaped relationship between vitamin A levels and health outcomes [35,36]. A meta-analysis showed that vitamin A supplementation significantly reduces inflammatory factors TNF-α and IL-6, lowering the risk of inflammatory diseases [35]. Short-term, low-dose vitamin A supplementation can raise plasma levels in certain groups, but this is not directly linked to a reduced protective effect against disease [35]. Furthermore, the wide 95% confidence intervals in some dose–response associations were due to small sample sizes, and the right-skewed distribution of blood vitamin levels supported this.

#### 4.1.3. The Interaction Effect of Age

We found that the protective effect of higher blood vitamin levels was not as significant as that of the group with lower levels, and the protective effect of vitamins against age-related diseases weakened with age. Wu et al. reported a negative correlation between dietary vitamin A intake and coronary artery disease prevalence [37], while Tang et al. found a similar correlation regarding cardiovascular metabolic coexistence [38]. These results differ from ours, likely due to the focus on dietary intake, whereas we measured blood vitamin levels. Physiological functions decline with age, affecting the body’s ability to synthesize and utilize vitamins [39]. Older individuals may also have lower vitamin levels due to reduced activity, insufficient sunlight, and an unbalanced diet [39]. Additionally, the risk of age-related diseases increases significantly with age, which may not be directly related to vitamin protective effects.

#### 4.1.4. Mediating Effect of BRI and Physical Activity

Our results showed that obesity can weaken the protective effects of vitamin A and B5 on HTN and DYS. A study in China indicated a correlation between serum vitamin A levels and obesity/metabolic syndrome in school-age children [40]. Obese individuals often experience heightened inflammatory responses and insulin resistance, which may impair the absorption and utilization of vitamins A and B5, thereby reducing their protective effects [41]. Conversely, physical activity can enhance the protective effects of vitamin B5 on DYS and HTN. Brancaccio et al. emphasized that combining physical activity with proper nutrition is crucial for preventing cardiovascular disease [42]. Physical activity increases energy expenditure, improves nutrient utilization efficiency, enhances cardiovascular function, and improves endothelial function, all of which facilitate decreasing the risk of cardiovascular disease [43].

### 4.2. Implication

The World Health Organization (WHO) emphasizes the importance of assessing nutritional status, including vitamin levels, to understand population health [44]. This study adheres to WHO guidelines to evaluate the blood vitamin status of adults in the PRD and generates corresponding frequency histograms, which help identify vitamin deficiencies and excess intakes that may impact health [44]. Based on our findings of J-shaped dose–response associations between vitamins and the risk of age-related diseases, this study can also serve as a baseline for monitoring changes in blood vitamin status and age-related diseases over time. This is particularly important in the PRD, which is experiencing rapid urbanization and lifestyle changes that may affect the nutritional status of its residents [45]. Additionally, factors such as age, obesity, and physical activity may play significant roles in the association between vitamins and age-related diseases. These findings remind health practitioners that they should offer tailored methods for individuals based on patients’ physiological characteristics, along with customized dietary plans [46]. An excessively high blood level of vitamins does not reduce the risk of age-related diseases, indicating that vitamin supplements are not recommended unless necessary. This cross-sectional study can serve as a foundation for future longitudinal or intervention studies that examine the temporal relationship between vitamin status and the onset of age-related diseases or explore the effectiveness of vitamin interventions [47].

### 4.3. Limitations

This study has several limitations. First, since the data are derived from a cross-sectional survey, it is impossible to determine whether other risk factors existed prior to the onset of age-related diseases [48]. Consequently, the results may be biased when compared to those from cohort studies. Second, this study examines the concentration of water-soluble vitamins in the blood, excluding dietary intake. Due to the lack of established standards for defining deficiencies in these vitamins, the study does not consider blood deficiencies [49]. Furthermore, Current clinical thresholds for water-soluble vitamins are inconsistent, complicating the classification of deficiencies. To address this issue, we employed quartile-based analyses (Q1–Q4) to investigate dose–response relationships instead of relying on binary deficiency categories. We emphasize that future studies should prioritize the harmonization of deficiency criteria through international collaborations. Finally, this study was conducted within the context of the PRD, which may limit its generalizability. However, many insights can still be applied to other contexts, particularly regarding vitamin interventions aimed at reducing the risk of age-related diseases in the population.

Despite the above limitations, this study also has several strengths. To our knowledge, it is the first investigation into the vitamin status of residents in the PRD, thereby providing new insights into this research area. We employed RCS to comprehensively elucidate the dose–response association between nine vitamins and five age-related diseases. Additionally, to further examine whether this association is influenced by other factors, we analyzed the mediating effects of various common lifestyle factors and conducted an interaction analysis based on age. Furthermore, the vitamin status data from the samples in this study were measured using precise blood biochemistry tests, eliminating self-report bias and thereby enhancing the reliability of the results.

## 5. Conclusions

We investigate the associations between vitamin status and age-related diseases among adults in the PRD region of China and address a significant gap in existing literature regarding multivitamin nutritional status. A J-shaped relationship was observed between vitamin levels and the risk of age-related diseases, except for the Vitamin A-HTN/T2DM. As the population ages, the burden of age-related diseases rises. Based on this scenario, this study emphasizes the need to assess vitamin status for public health and aims to guide future research and health interventions.

## Figures and Tables

**Table 1:** Characteristics of participants.

Characteristics	Total	CVD	*p*-Value	CHD	*p*-Value	HTN	*p*-Value	DYS	*p*-Value	T2DM	*p*-Value
**Age** (mean ± SD, years)	50.92 ± 9.30	61.66 ± 10.76	**<0.001**	65.48 ± 12.70	**<0.001**	54.87 ± 10.62	**<0.001**	53.72 ± 9.12	**<0.001**	58.94 ± 10.40	**<0.001**
**Sex** (n, %)											
Female	951 (35.94)	36 (2.13)	0.966	22 (1.30)	0.229	368 (21.71)	**0.026**	286 (16.87)	**0.036**	103 (6.08)	0.330
Male	1695 (64.06)	20 (2.15)		18 (2.00)		172 (18.09)		131 (13.78)		67 (7.05)	
**Education** (n, %)											
Primary School and Below	307 (11.60)	16 (5.25)	**<0.001**	10 (3.26)	**0.048**	91 (29.64)	**<0.001**	37 (12.05)	**<0.001**	44 (14.33)	**<0.001**
Middle School	885 (33.45)	20 (2.27)		8 (0.90)		156 (17.63)		75 (8.48)		45 (5.09)	
High School	990 (37.41)	8 (0.81)		16 (1.62)		200 (20.20)		168 (16.98)		58 (5.86)	
Undergraduate	434 (16.40)	12 (2.77)		5 (1.15)		88 (20.28)		125 (28.80)		22 (5.07)	
Postgraduate and Above	30 (1.13)	0 (0.00)		1 (3.33)		5 (16.67)		12 (40.00)		1 (3.33)	
**BRI** ^a^ (n, %)											
<3.41 (Very thin)	2590 (97.88)	55 (2.13)	0.990	38 (1.47)	0.552	521 (2.01)	**0.016**	405 (15.64)	0.621	1665.41)	0.916
3.41–4.44 (Thin)	51 (1.93)	1 (1.96)		2 (3.92)		19 (37.26)		11 (21.57)		4 (7.84)	
4.45–5.45 (Normal)	4 (0.15)	0 (0.00)		0 (0.00)		0 (0.00)		1 (25.00)		0 (0.00)	
>5.46 (Mellow)	1 (0.04)	0 (0.00)		0 (0.00)		0 (0.00)		0 (0.00)		0 (0.00)	
**Smoking** (n, %)	642 (24.26)	14 (2.19)	0.892	7 (1.09)	0.315	116 (18.07)	0.091	91 (14.17)	0.205	32 (4.98)	0.087
**Alcohol** **Consumption** (n, %)	1026 (38.78)	10 (0.98)	**0.003**	8 (0.78)	**0.031**	188 (18.32)	**<0.001**	164 (15.99)	0.033	52 (5.07)	**0.018**
**Physical** **inactivity** (n, %)	1075 (40.63)	20 (1.86)	0.444	14 (1.30)	0.465	237 (22.05)	0.084	187 (17.40)	0.056	64 (5.95)	0.413
**Family history of disease** (n, %)	-	14 (5.28)	**<0.001**	8 (4.37)	**0.001**	277 (34.62)	**<0.001**	60 (14.42)	**0.004**	51 (30.18)	**<0.001**
**Fasting blood glucose** (mmol/L)	4.87 ± 1.55	5.52 ± 2.31	0.217	5.46 ± 1.40	0.108	5.14 ± 1.80	0.804	4.99 ± 1.41	0.980	6.74 ± 2.56	**<0.001**
**Glycosylated hemoglobin** (%)	5.25 ± 0.81	5.52 ± 0.87	0.298	5.43 ± 0.74	0.583	5.33 ± 0.90	0.702	5.33 ± 0.76	0.714	6.21 ± 1.45	**<0.001**
**Triglyceride** (mmol/L)	1.49 ± 0.84	1.50 ± 0.70	0.803	1.48 ± 0.80	0.917	1.64 ± 0.88	0.264	1.84 ± 0.96	**<0.001**	1.58 ± 0.82	0.429
**Total cholesterol** (mmol/L)	4.55 ± 0.93	4.30 ± 1.04	0.312	4.41 ± 0.93	0.687	4.58 ± 0.98	0.109	4.80 ± 0.99	**<0.001**	4.51 ± 1.03	0.201
**LDL-C** (mmol/L)	2.44 ± 1.04	2.42 ± 0.95	0.517	2.52 ± 0.91	0.323	2.60 ± 0.81	0.103	2.78 ± 0.95	**<0.001**	2.59 ± 0.85	0.900
**HDL-C** (mmol/L)	1.25 ± 0.32	1.24 ± 0.43	0.826	1.20 ± 0.25	0.472	1.19 ± 0.26	0.269	1.18 ± 0.29	0.106	1.20 ± 0.26	0.399
**Homocysteine** (mol/L)	13.58 ± 6.91	13.83 ± 5.69	**<0.001**	14.22 ± 8.54	**<0.001**	14.14 ± 6.68	**<0.001**	13.65 ± 6.93	0.067	14.48 ± 8.64	**<0.001**

^a^ According to anonymized data from bri-calculator.net, the average Body Resilience Index (BRI) of the Chinese population is 3.34. Currently, there is no authoritative standard for classifying BRI in China; the classification is based on existing international standards. *BRI:* Body Roundness Index; *SD:* Standard deviation; *LDL-C:* Low-Density Lipoprotein Cholesterol; *HDL-C:* High-Density Lipoprotein Cholesterol. *CVD:* Cerebrovascular Disease; *CHD:* Coronary Heart Disease; *HTN:* Hypertension; *DYS:* Dyslipidemia; *T2DM:* Type-2 Diabetes Mellitus.

**Table 2:** The Mediating Role of the BRI, Smoking, Alcohol Consumption, and Physical Activity in the Association Between Vitamin Status and Age-Related Diseases.

	Total Effect (95%CI)	Direct Effect (95% CI)	Indirect Effect (95% CI)	pMe (%) ^a^
**Vitamin A—HTN**				
BRI	0.200 (0.106, 0.294) ***	0.161 (0.065, 0.255) ***	0.039 (0.022, 0.056) ***	**19.73**
Smoking	0.191 (0.099, 0.287) ***	0.193 (0.099, 0.287) ***	−0.002 (−0.008, 0.003)	-
Alcohol	0.190 (0.097, 0.283) ***	0.193 (0.100, 0.287) ***	−0.003 (−0.013, 0.006)	-
Physical Activity	0.186 (0.092, 0.280) ***	0.190 (0.096, 0.284) ***	−0.003 (−0.010, 0.004)	-
**Vitamin A—DYS**				
BRI	0.234 (0.132, 0.335) ***	0.205 (0.103, 0.307) ***	0.029 (0.013, 0.044) ***	**12.35**
Smoking	0.231 (0.130, 0.332) ***	0.233 (0.132, 0.334) ***	−0.002 (−0.006, 0.003)	-
Alcohol	0.230 (0.128, 0.330) ***	0.230 (0.129, 0.331) ***	−0.008 (−0.011, 0.010)	-
Physical Activity	0.228 (0.126, 0.330) ***	0.231 (0.129, 0.332) ***	−0.002 (−0.008, 0.003)	-
**Vitamin A—T2DM**				
BRI	0.144 (−0.007, 0.296)	0.129 (−0.023, 0.282)	0.015 (−0.004, 0.034)	-
Smoking	0.140 (−0.011, 0.292)	0.143 (−0.008, 0.295)	−0.003 (−0.010, 0.004)	-
Alcohol	0.140 (−0.011, 0.291)	0.141 (−0.011, 0.292)	−0.001 (−0.017, 0.015)	-
Physical Activity	0.130 (−0.023, 0.284)	0.132 (−0.022, 0.285)	−0.001 (−0.006, 0.003)	-
**Vitamin B_1_—CVD**				
BRI	0.045 (−0.131, 0.221)	0.042 (−0.134, 0.219)	0.002 (−0.005, 0.010)	-
Smoking	0.004 (−0.130, 0.216)	0.046 (−0.127, 0.219)	−0.003 (−0.012, 0.006)	-
Alcohol	0.045 (−0.133, 0.224)	0.041 (−0.138, 0.219)	0.005 (−0.008, 0.017)	-
Physical Activity	0.140 (−0.111, 0.391)	0.157 (−0.094, 0.408)	−0.017 (−0.046, 0.012)	-
**Vitamin B_1_—HTN**				
BRI	0.023 (−0.073, 0.120)	0.015 (−0.082, 0.111)	0.008 (−0.007, 0.024)	-
Smoking	0.021 (−0.075, 0.118)	0.018 (−0.078, 0.115)	0.003 (−0.002, 0.008)	-
Alcohol	0.022 (−0.074, 0.118)	0.022 (−0.074, 0.118)	0.0002 (−0.002, 0.002)	-
Physical Activity	0.070 (−0.051, 0.191)	0.086 (−0.035, 0.208)	−0.016 (−0.030,−0.003)	-
**Vitamin B_5_—CVD**				
BRI	0.110 (−0.123, 0.343)	0.096 (−0.141, 0.333)	0.014 (−0.030, 0.058)	-
Smoking	0.110 (−0.124, 0.344)	0.116 (−0.120, 0.351)	−0.004 (−0.020, 0.008)	-
Alcohol	0.107 (−0.127, 0.340)	0.105 (−0.129, 0.338)	0.002 (−0.009, 0.013)	-
Physical Activity	0.087 (−0.151, 0.326)	0.101 (−0.137, 0.339)	−0.014 (−0.038, 0.010)	-
**Vitamin B_5_—HTN**				
BRI	0.178 (0.078, 0.279) ***	0.123 (0.022, 0.225) *	0.055 (0.034, 0.076) ***	**30.81**
Smoking	0.172 (0.079, 0.271) ***	0.167 (0.068, 0.265) ***	0.005 (−0.0009, 0.012)	-
Alcohol	0.172 (0.073, 0.270) ***	0.172 (0.074, 0.270) ***	0.0008 (−0.0007, 0.0009)	
Physical Activity	0.177 (0.077, 0.276) ***	0.191 (0.090, 0.291) ***	−0.014 (−0.025, 0.003) *	**−8.10**
**Vitamin B_5_—DYS**				
BRI	0.233 (0.127, 0.340) ***	0.194 (0.087, 0.301) ***	0.039 (0.019, 0.059) ***	**16.80**
Smoking	0.229 (0.123, 0.334) ***	0.225 (0.119, 0.330) ***	0.004 (−0.002, 0.010)	-
Alcohol	0.228 (0.123, 0.334) ***	0.229 (0.123, 0.334) ***	−0.0008 (−0.0009, 0.0007)	-
Physical Activity	0.242 (0.136, 0.348) ***	0.254 (0.148, 0.361) ***	−0.012 (−0.023,−0.001) *	**−5.07**
**Vitamin B_6_—CVD**				
BRI	−0.117 (−0.470, 0.236)	−0.116 (−0.469, 0.237)	−0.001 (−0.006, 0.004)	-
Smoking	−0.120 (−0.474, 0.234)	−0.131 (−0.487, 0.225)	0.011 (−0.015, 0.037)	-
Alcohol	−0.125 (−0.477, 0.228)	−0.118 (−0.471, 0.234)	−0.006 (−0.020, 0.007)	-
Physical Activity	−0.130 (−0.495, 0.236)	−0.146 (−0.512, 0.220)	0.016 (−0.012, 0.044)	-

This mediated utility model was analyzed using Logit regression and subjected to the KHB test. The model was adjusted for sex (female or male), age (years, continuous), education (Primary School and Below/Middle School/High School/Undergraduate/Postgraduate and Above), family history of disease (yes or no, corresponds to 5 diseases) and biochemical indicators (CVD, CHD and HTN: homocysteine levels; DYS: Triglycerides, total cholesterol and LDL-C; T2DM: Fasting blood glucose, homocysteine levels and glycosylated hemoglobin). ^a^ pMe is the proportion of the total effect that is mediated. * *p* < 0.05, ** *p* < 0.01, *** *p* < 0.001 ***CVD:*** Cerebrovascular Disease; ***CHD:*** Coronary Heart Disease; ***HTN:*** Hypertension; ***DYS:*** Dyslipidemia; ***T2DM:*** Type-2 Diabetes Mellitus.

## Data Availability

The data are not publicly available due to Zhuhai People’s Hospital’s policy and ethical considerations. However, the data presented in this study are available upon request from the corresponding author.

## Supplementary Materials

The following supporting information can be downloaded at: https://www.mdpi.com/article/10.3390/nu17101637/s1, Supplemental Table S1: STROBE Statement—Checklist of items that should be included in reports of cross-sectional studies; Supplemental Table S2: Sample Quality Test Form; Supplemental Table S3: Definition Rule of age-related disease factors; Supplemental Table S4: Distribution of blood vitamin concentrations in the study population; Supplemental Table S5: Fat-soluble Vitamin status of participants grouped by sex and age; Supplemental Table S6: Water-soluble Vitamin status of participants grouped by sex and age; Supplemental Table S7. Odds ratios (95% CIs) of vitamin status with age-related diseases; Supplemental Table S8: Age as an Interaction Factor to Explore Logit Models for Vitamins and Age-Related Diseases; Supplemental Figure S1: Blood Vitamin Levels in Adults in the Pearl River Delta Region; Supplemental Figure S2: Sensitivity Analysis of Restricted Cubic Splines; Supplemental Figure S3: Performance evaluation (ROC curve) of the fully adjusted logistic model used for the RCS method.

## Abbreviations

The following abbreviations are used in this manuscript:

PRD	Pearl River Delta
HbA1c	Glycosylated hemoglobin
LDL-C	Low-Density Lipoprotein Cholesterol
HDL-C	High-Density Lipoprotein Cholesterol
Cys	Homocysteine
BRI	Body Roundness Index
SD	Standard deviation
CI	Confidence intervals
RCS	Restricted Cubic Spline
OR	Odds ratio
CVD	Cerebrovascular Disease
CHD	Coronary Heart Disease
HTN	Hypertension
DYS	Dyslipidemia
T2DM	Type-2 Diabetes Mellitus
