# Supplementary material for: Vitamin Status and Risk of Age-Related Diseases Among Adult Residents of the Pearl River Delta Region
Source: Nutrients. 2025 May 10;17(10):1637. doi: 10.3390/nu17101637 (PMC12114280; doi:10.3390/nu17101637)

Vitamin Status and Risk of Age-Related Diseases Among Adult Residents of the Pearl River Delta Region

Supplementary

1 Additional file S1. STROBE checklist 2

1.1 Supplemental Table S1. STROBE Statement—Checklist of items that should be included in reports of cross-sectional studies 2

2 Additional file S2. Measurement of Vitamin A, B, D, and E 4

2.1 Supplemental Table S2 Sample Quality Test Form 5

3 Additional file S3. Definition of Covariates 6

3.1 Supplemental Table S3. Definition Rule of age-related disease factors 6

4 Additional file S4. Distribution of vitamins in participants 7

4.1 Supplemental Table S4. Distribution of blood vitamin concentrations in the study population 7

4.2 Supplemental Table S5. Fat-soluble Vitamin status of participants grouped by sex and age 7

4.3 Supplemental Table S6. Water-soluble Vitamin status of participants grouped by sex and age 8

4.4 Supplemental Figure S1. Blood Vitamin Levels in Adults in the Pearl River Delta Region 9

5 Additional file S5. Association between blood vitamin concentrations and age-related diseases 10

5.1 Supplemental Table S7. Odds ratios (95% CIs) of vitamin status with age-related diseases 10

6 Additional file S6. Age as an interaction term to analyze the association between vitamins and age-related diseases 17

6.1 Supplemental Table S8. Age as an Interaction Factor to Explore Logit Models for Vitamins and Age-Related Diseases 17

7 Additional file S7. Results of sensitivity analysis 18

7.1 Supplemental Figure S2. Sensitivity Analysis of Restricted Cubic Splines 18

7.1.1 A Equidistant knots (P30,P60,P90) 18

7.1.2 B Four knots (P_{20}, P_{40}, P_{60}, P_{80}) 19

7.2 Supplemental Figure S3. Performance evaluation (ROC curve) of the fully adjusted logistic model used for the RCS method 20

1 Additional file S1. STROBE checklist

1.1 Supplemental Table S1. STROBE Statement—Checklist of items that should be included in reports of cross-sectional studies

	Item No	Recommendation	Page No
**Title and abstract**	1	(*a*) Indicate the study's design with a commonly used term in the title or the abstract	1-2
		(*b*) Provide in the abstract an informative and balanced summary of what was done and what was found	1-2
**Introduction**			
Background/rationale	2	Explain the scientific background and rationale for the investigation being reported	2
Objectives	3	State specific objectives, including any prespecified hypotheses	2
**Methods**			
Study design	4	Present key elements of study design early in the paper	3-4
Setting	5	Describe the setting, locations, and relevant dates, including periods of recruitment, exposure, follow-up, and data collection	3-4
Participants	6	(*a*) Give the eligibility criteria, and the sources and methods of selection of participants.	3-4
		(*b*) For matched studies, give matching criteria and number of exposed and unexposed	-
Variables	7	Clearly define all outcomes, exposures, predictors, potential confounders, and effect modifiers. Give diagnostic criteria, if applicable	3-5
Data sources/ measurement	8*	For each variable of interest, give sources of data and details of methods of assessment (measurement). Describe comparability of assessment methods if there is more than one group	3
Bias	9	Describe any efforts to address potential sources of bias	3-5, 13-14
Study size	10	Explain how the study size was arrived at	3
Quantitative variables	11	Explain how quantitative variables were handled in the analyses. If applicable, describe which groupings were chosen and why	3-5
Statistical methods	12	(*a*) Describe all statistical methods, including those used to control for confounding	5-6
		(*b*) Describe any methods used to examine subgroups and interactions	5-6
		(*c*) Explain how missing data were addressed	5-6
		(*d*) If applicable, describe analytical methods taking account of sampling strategy	3
		(*e*) Describe any sensitivity analyses	6
**Results**			
Participants	13*	(a) Report numbers of individuals at each stage of study—eg numbers potentially eligible, examined for eligibility, confirmed eligible, included in the study, completing follow-up, and analysed	6-7
		(b) Give reasons for non-participation at each stage	6-8
		(c) Consider use of a flow diagram	4
Descriptive data	14*	(a) Give characteristics of study participants (eg demographic, clinical, social) and information on exposures and potential confounders	6-8

		(b) Indicate number of participants with missing data for each variable of interest	-
Outcome data	15*	Report numbers of outcome events or summary measures	8-11
Main results	16	(a) Give unadjusted estimates and, if applicable, confounder-adjusted estimates and their precision (eg, 95% confidence interval). Make clear which confounders were adjusted for and why they were included	8-11
		(b) Report category boundaries when continuous variables were categorized	8-11
		(c) If relevant, consider translating estimates of relative risk into absolute risk for a meaningful time period	8-11
Other analyses	17	Report other analyses done—eg analyses of subgroups and interactions, and sensitivity analyses	8-11
**Discussion**			
Key results	18	Summarise key results with reference to study objectives	11-12
Limitations	19	Discuss limitations of the study, taking into account sources of potential bias or imprecision. Discuss both direction and magnitude of any potential bias	13
Interpretation	20	Give a cautious overall interpretation of results considering objectives, limitations, multiplicity of analyses, results from similar studies, and other relevant evidence	13-14
Generalisability	21	Discuss the generalisability (external validity) of the study results	11-13
**Other information**			
Funding	22	Give the source of funding and the role of the funders for the present study and, if applicable, for the original study on which the present article is based	14

*Give information separately for exposed and unexposed groups.

Note: An Explanation and Elaboration article discusses each checklist item and gives methodological background and published examples of transparent reporting. The STROBE checklist is best used in conjunction with this article (freely available on the Web sites of PLoS Medicine at http://www.plosmedicine.org/, Annals of Internal Medicine at http://www.annals.org/, and Epidemiology at http://www.epidem.com/). Information on the STROBE Initiative is available at http://www.strobe-statement.org.

Additional file S2. Measurement of Vitamin A, B, D, and E

The study population consisted of community residents, and factors such as convenience, comfort, cost-effectiveness, sample size, and flexibility were considered when selecting fingertip-collected whole blood samples for the dried blood spot (DBS) card. The test sample type utilized was whole blood. The dried blood spot (DBS) method of blood sample collection has gained popularity in clinical research, particularly in situations where conventional blood sampling is challenging. DBS offers a less invasive, easy-to-perform, and cost-effective approach to sample collection. Recent studies have demonstrated the reliability and utility of DBS in measuring vitamin levels. For instance, Nybo et al. (2021) showed that vitamin D measurements in DBS samples were not significantly different from those in venous plasma samples, even under varying temperature conditions. Additionally, Huang et al. (2020) further validated the accuracy and stability of DBS in assessing vitamin B1 (thiamine) status. Binks et al. developed a dry blood spot (DBS)-based liquid chromatography-tandem mass spectrometry (LC-MS/MS) method for measuring 25-hydroxyvitamin D (25OHD) metabolites. This method demonstrated the accuracy of DBS sampling for assessing vitamin D levels and its applicability across various environmental conditions, making it a valuable tool for evaluating vitamin D levels in resource-limited settings. Zakaria et al. introduced a candidate reference method for vitamin D determination in DBS samples, which further enhances the accuracy and traceability of vitamin D measurements in this format (Binks, Bleakley, Rathnayake, Pizzutto, Chang, McWhinney, & Ungerer, 2021; Zakaria, Allen, Koplin, Roche, & Greaves, 2020). Collectively, these findings support the use of DBS as a reliable and practical approach for measuring vitamin D levels in clinical and epidemiological studies.

Dried blood spot (DBS) samples were prepared as follows: subjects fasted for 10 to 12 hours, after which fasting whole blood was collected in the early morning of the following day. A total of 5 mL of fasting whole blood was collected, placed in EDTA anticoagulation tubes, and stored in an insulated box for short-term refrigeration. The samples were then sent to the laboratory for the preparation of dried blood spot cards within six hours. The preparation process involved pipetting 20 μL of whole blood and placing it in the center of the marking circle on the DBS card to create dried blood spot cards for vitamin testing. After drying, the card was placed in a sealed aluminum foil bag and stored in a refrigerator at -80°C for subsequent vitamin analysis. The dried blood spot cards were prepared in a biosafety cabinet and were protected from light.

Since the general definition of fat-soluble vitamin deficiency or insufficiency is based on serum samples, both serum and whole blood samples from 150 individuals were tested during the pre-study phase of this research. Vitamin levels were converted to entire blood fat-soluble vitamin definitions based on serum determinations as follows: vitamin A <297.8 ng/mL, vitamin D <16.5 ng/mL (men) or 17.7 ng/mL (women), vitamin E <3.42 μmol (women), and vitamin K <1.5 ng/mL (men) and <3.5 ng/mL (women). Additionally, vitamin E was defined as <3.42 μg/mL. The methodological validation of LC-MS/MS in this study was based on the European Medicines Agency (EMA) Guidelines for the Validation of Analytical Methods, with key parameters including the Lower Limit of Quantitation (LLoQ), accuracy, precision, and spiked recovery. During sample testing and analysis, three quality control (QC) samples were included in each batch of 60 samples for monitoring purposes, and the intra-day and inter-day coefficients of variation were maintained at <10% (see Supplemental Table 2).

2.1 Supplemental Table S2 Sample Quality Test Form

Targets	Spiked concentration (ng/mL)±SD	Intra-day (n=6)		Inter-day (n=6)	
		Measured concentration (ng/mL)±SD	Recoveries% (CV%)	Measured concentration (ng/mL)±SD	Recoveries% (CV%)
**Thiamine**					
LLOQ-level	1.0	0.96±0.10	96.0 (10.4)	0.97±0.10	97.0 (10.3)
Low-level	5.0	4.70±0.33	94.0 (7.0)	5.01±0.38	100.0 (7.6)
Medium-level	20.0	18.91±0.70	94.6 (3.7)	18.92±0.98	94.6 (5.2)
High-level	40.0	38.40±1.22	96.0 (3.2)	39.40±0.98	98.5 (2.4)
**Riboflavin**					
LLOQ-level	0.5	0.46±0.07	92.0 (15.2)	0.49±0.08	98.0 (16.3)
Low-level	5.0	4.82±0.44	96.4 (9.1)	4.69±0.30	93.8 (6.4)
Medium-level	20.0	18.68±1.19	93.4 (6.4)	18.79±1.18	94.0 (6.3)
High-level	40.0	37.75±1.15	94.4 (3.0)	37.87±1.95	94.6 (5.1)
**Nicotimamide**					
LLOQ-level	0.5	0.48±0.10	96.0 (20.8)	0.59±0.09	118.0 (15.3)
Low-level	5.0	5.57±0.76	111.4 (13.6)	5.50±0.42	110.0 (7.6)
Medium-level	20.0	19.01±0.41	95.1 (2.2)	20.05±0.82	100.3 (4.1)
High-level	40.0	38.99±3.35	97.5 (8.6)	39.50±2.46	98.8 (6.2)
**Pantothenic Acid**					
LLOQ-level	1.0	1.13±0.06	113.0 (5.3)	1.02±0.15	102.0 (14.7)
Low-level	5.0	5.16±0.35	103.2 (6.8)	5.14±0.61	102.8 (11.9)
Medium-level	20.0	20.08±0.91	100.4 (4.5)	20.22±1.02	101.1 (5.0)
High-level	40.0	39.30±1.22	98.3 (3.1)	40.72±1.69	101.8 (4.2)
**Pyridoxine**					
LLOQ-level	0.5	0.50±0.06	100.0 (12.0)	0.46±0.06	92.0 (13.0)
Low-level	5.0	4.77±0.37	95.4 (7.8)	4.47±0.28	89.4 (6.3)
Medium-level	20.0	18.78±0.91	93.9 (4.8)	18.34±1.27	91.7 (6.9)
High-level	40.0	37.82±1.15	94.6 (3.0)	36.86±3.27	92.2 (8.9)
**Folic Acid**					
LLOQ-level	0.5	0.59±0.09	118.0 (15.3)	0.48±0.08	96.0 (16.7)
Low-level	5.0	4.81±0.31	96.2 (6.4)	4.74±0.36	94.8 (7.6)
Medium-level	20.0	18.74±0.77	93.7 (4.1)	18.35±1.05	91.8 (5.7)
High-level	40.0	37.83±1.41	94.6 (3.7)	37.76±1.52	94.4 (4.0)
**Retinol**					
LLOQ-level	20.0	17.74±1.68	88.7 (9.5)	18.26±2.15	91.3 (11.8)
Low-level	50.0	46.83±5.67	93.7 (12.1)	52.27±5.96	104.5 (11.4)
Medium-level	500.0	468.77±33.26	93.8 (7.1)	524.33±35.37	104.9 (6.7)
High-level	2000.0	2133.02±71.85	106.6 (3.4)	2169.32±93.96	108.5 (4.3)
**25-OHD$_2$**					
LLOQ-level	2.0	1.99±0.30	99.5 (15.1)	1.83±0.33	91.5 (18.0)
Low-level	5.0	4.88±0.59	97.6 (12.1)	4.57±0.65	91.4 (14.2)
Medium-level	20.0	19.36±2.18	96.8 (11.3)	18.98±1.94	94.9 (10.2)
High-level	40.0	38.47±2.66	96.2 (6.9)	39.46±2.53	98.7 (6.4)
**25-OHD$_3$**					
LLOQ-level	0.5	0.42±0.05	84.0 (11.9)	0.45±0.08	90.0 (17.8)
Low-level	5.0	5.41±0.60	108.2 (11.1)	5.24±0.49	104.8 (9.4)
Medium-level	20.0	21.14±1.12	105.7 (5.3)	18.90±1.50	94.5 (7.9)
High-level	40.0	42.65±2.02	106.4 (4.7)	39.51±1.76	98.9 (4.5)
**α-Tocopherol**					
LLOQ-level	400.0	367.28±28.67	91.8 (7.8)	406.60±45.29	101.7 (11.1)
Low-level	1000.0	912.61±81.05	91.3 (8.9)	918.61±48.31	91.8 (5.3)
Medium-level	4000.0	4251.35±113.31	106.3 (2.7)	3908.38±194.79	97.7 (5.0)
High-level	8000.0	8270.17±613.24	103.4 (7.4)	7481.78±600.19	93.5 (8.0)

SD Standard deviation

3 Additional file S3. Definition of Covariates

3.1 Supplemental Table S3. Definition Rule of age-related disease factors

	Variables	Definition	Tyoe
1	Age	The real age is calculated by subtracting the respondent's birth year from the interview year.	Continuous variable in years.
2	Sex	The gender status of the respondent.	Female / Male
3	Education level	The highest level of education attained by the respondents was reported by themselves.	Primary School and Below/ Middle School/ High School/ Undergraduate/ Postgraduate and Above
4	BRI	The physical examination is conducted by a medical professional using a tape measure to measure height, weight and waist circumference, which is calculated according to a formula.	We categorize them when descriptive statistics are performed and we treat them as continuous variables when modeling regression.<br>< 3.41 (Very thin) / 3.41-4.44 (Thin) / 4.45-5.45 (Normal) / 5.46-6.91 (Mellow) / > 6.91 (Very Mellow)
5	Smoking	Respondents are classified based on their smoking status.	No / Yes /discontinued smoking<br>Mediated effects were analyzed by treating smoking cessation as No
6	Alcohol consumption	Respondents are classified based on their drinking status.	No / Yes / discontinued drinking<br>Mediated effects were analyzed by treating drinking cessation as No
7	Physical activity	From participant self-report, regular exercise was standardized as moderate-intensity exercise equivalent to brisk walking and greater than 3 times per week for 30 minutes or more, including moderate and heavy physical workers.	No / Yes
8	Family history of disease	This information was first provided by case material, or self-reported by the participant if the participant was unable to provide case material, or if there was a family history of the disease in question, the relationship of the family member with the disease to the person, and the number of people with the disease.	No / Yes
9	Fasting blood glucose	Biochemical indicators, in accordance with the technical specifications for laboratory testing.	Continuous variable (mmol/L)
10	Glycosylated hemoglobin		Continuous variable (%)
11	Triglyceride		Continuous variable (mmol/L)
12	Total cholesterol		Continuous variable (mmol/L)
13	Low-Density Lipoprotein Cholesterol		Continuous variable (mmol/L)
14	High-Density Lipoprotein Cholesterol		Continuous variable (mmol/L)
15	Homocysteine		Continuous variable (mol/L)

4 Additional file S4. Distribution of vitamins in participants

4.1 Supplemental Table S4. Distribution of blood vitamin concentrations in the study population

Variables	Percentile			Min	Max
	25th	50th	75th		
Vitamin A (ng/mL)	312.90	367.70	465.20	134.00	1389.00
Vitamin D (ng/mL)	23.43	33.00	45.46	7.39	99.83
Vitamin E (μg/mL)	3.50	4.26	5.48	1.44	18.79
Vitamin B_1 (ng/mL)	1.47	3.43	7.00	0.23	182.30
Vitamin B_2 (ng/mL)	4.04	10.06	28.45	0.11	320.00
Vitamin B_3 (μg/mL)	2.21	2.79	3.64	0.93	19.61
Vitamin B_5 (ng/mL)	123.20	165.10	223.60	23.90	989.00
Vitamin B_6 (ng/mL)	4.94	11.18	17.26	1.02	110.10
Vitamin B_9 (ng/mL)	2.00	3.72	6.03	0.10	20.60

4.2 Supplemental Table S5. Fat-soluble Vitamin status of participants grouped by sex and age

	Deficiency (%)	Normal (%)	Total	P-value*
**Vitamin A**				
Female	210 (22.06)	742 (77.94)	952	<0.001
Male	292 (17.20)	1406 (82.80)	1698	
Young	166 (23.55)	539 (76.45)	705	<0.001
Middle-aged	274 (18.48)	1209 (81.52)	1483	
Older	62 (13.42)	400 (86.58)	462	
**Vitamin D**				
Female	136 (14.29)	816 (85.71)	952	<0.001
Male	124 (7.30)	1574 (92.70)	1698	
Young	56 (7.94)	649 (92.06)	705	<0.001
Middle-aged	133 (8.97)	1350 (91.03)	1483	
Older	71 (15.37)	391 (84.63)	462	
**Vitamin E**				
Female	148 (15.55)	804 (84.45)	952	<0.001
Male	431 (25.38)	1267 (74.62)	1698	
Young	173 (24.54)	532 (75.46)	705	0.017
Middle-aged	334 (22.52)	1149 (77.48)	1483	
Older	72 (15.58)	390 (84.42)	462	

*P values of Chi-squared test.

4.3 Supplemental Table S6. Water-soluble Vitamin status of participants grouped by sex and age

Variables	Percentile			Min	Max	P-value*
	25th	50th	75th			
**Vitamin B_1 (ng/mL)**						
Female	3.58	7.32	11.33	0.41	182.30	<0.001
Male	1.21	2.24	4.60	0.23	75.23	
Young	1.21	2.05	4.42	0.35	48.48	
Middle-aged	1.38	2.95	6.33	0.23	75.23	<0.001
Older	5.41	7.75	11.21	0.43	182.30	
**Vitamin B_2 (ng/mL)**						
Female	10.155	24.46	44.62	0.43	320.00	<0.001
Male	3.01	6.33	15.84	0.11	315.20	
Young	2.76	5.85	12.83	0.11	223.30	
Middle-aged	3.90	8.92	24.23	0.18	240.70	<0.001
Older	18.73	33.38	52.31	0.76	320.00	
**Vitamin B_3 (µg/mL)**						
Female	2.24	2.80	3.53	0.97	17.41	0.676
Male	2.20	2.79	3.71	0.93	19.61	
Young	2.10	2.77	3.65	0.93	19.61	
Middle-aged	2.20	2.76	3.61	0.97	17.41	0.111
Older	2.36	2.88	3.64	1.09	12.77	
**Vitamin B_5 (ng/mL)**						
Female	146.65	199.90	267.95	23.90	885.70	<0.001
Male	118.30	150.65	194.10	32.14	989.00	
Young	113.40	139.10	178.20	23.90	885.70	
Middle-aged	122.90	160.70	209.20	32.14	989.00	<0.001
Older	191.20	247.80	315.00	41.75	961.40	
**Vitamin B_6 (ng/mL)**						
Female	3.71	6.04	12.65	1.02	69.62	<0.001
Male	7.76	13.44	18.91	1.02	110.10	
Young	9.16	13.62	18.65	1.03	110.10	
Middle-aged	5.82	12.14	18.09	1.02	109.10	<0.001
Older	2.89	4.19	6.91	1.02	69.62	
**Vitamin B_9 (ng/mL)**						
Female	1.86	3.44	5.81	0.10	20.33	<0.001
Male	2.12	3.88	6.11	0.11	20.60	
Young	2.02	3.82	6.13	0.11	20.60	
Middle-aged	2.16	3.88	6.20	0.10	20.33	0.004
Older	1.39	3.02	5.26	0.13	10.30	

*P values of Mann-Whitney U test.

4.4 Supplemental Figure S1. Blood Vitamin Levels in Adults in the Pearl River Delta Region

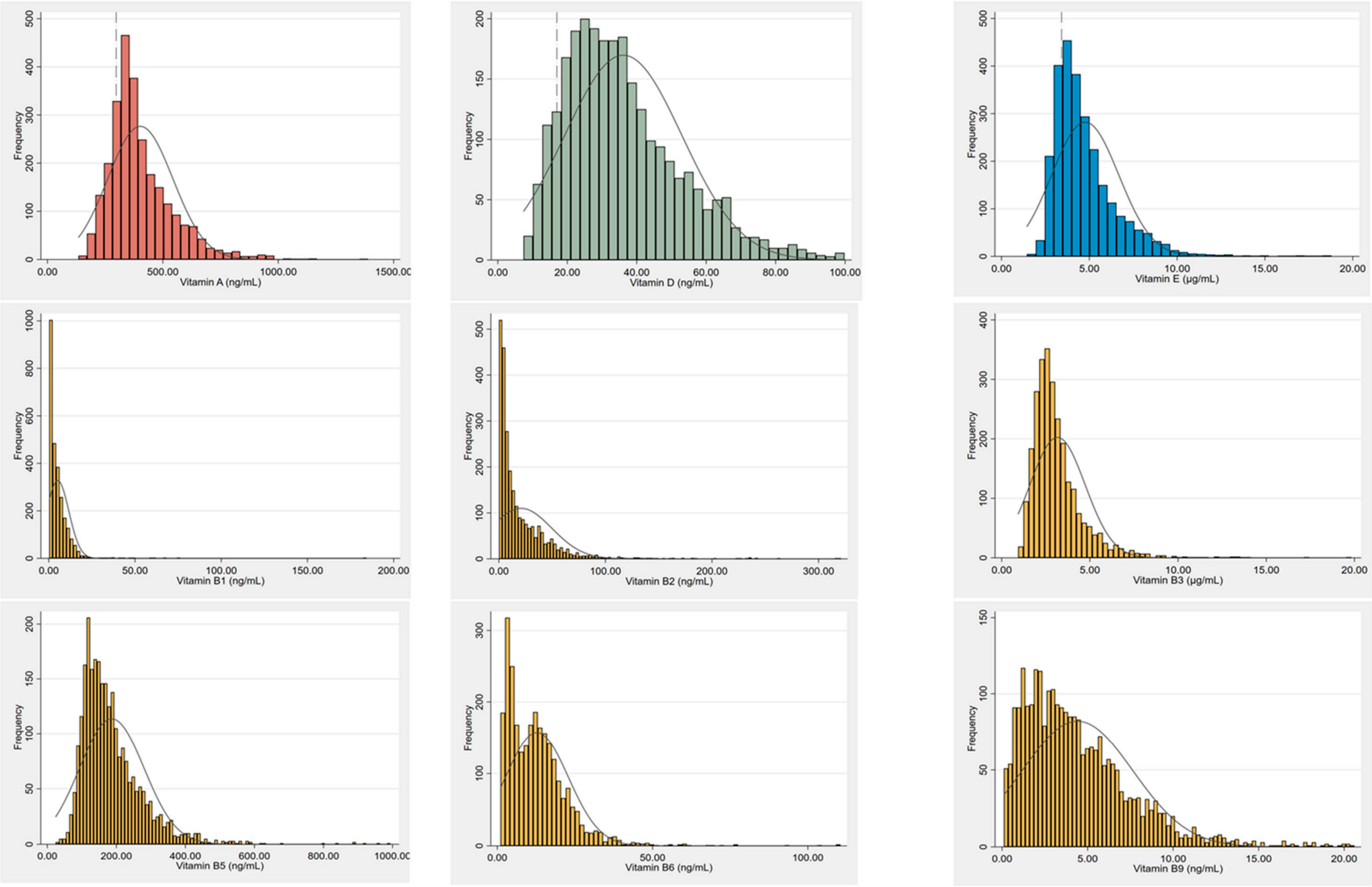

5 Additional file S5. Association between blood vitamin concentrations and age-related diseases

5.1 Supplemental Table S7. Odds ratios (95% CIs) of vitamin status with age-related diseases

Vitamin	Group		Quartile-based Group			
	Normal	Deficiency	Q_1	Q_2	Q_3	Q_4
**Vitamin A**						
CVD						
Model 1	Ref.	0.41(0.16,1.04)				
Model 2	Ref.	0.46(0.18,1.15)				
Model 3	Ref.	0.42(0.15,1.22)				
CHD						
Model 1	Ref.	0.75(0.31,1.80)				
Model 2	Ref.	0.67(0.23,1.93)				
Model 3	Ref.	0.55(0.16,1.86)				
HTN						
Model 1	Ref.	**0.63(0.48,0.82)*****				
Model 2	Ref.	**0.69(0.52,0.92)****				
Model 3	Ref.	**0.74(0.55,0.99)***				
DYS						
Model 1	Ref.	**0.57(0.40,0.75)*****				
Model 2	Ref.	**0.57(0.42,0.80)*****				
Model 3	Ref.	**0.59(0.42,0.81)*****				
T2DM						
Model 1	Ref.	**0.40(0.23,0.68)*****				
Model 2	Ref.	**0.51(0.29,0.86)****				
Model 3	Ref.	**0.51(0.29,0.90)****				
**Vitamin D**						
CVD						
Model 1	Ref.	1.10(0.47,2.60)				
Model 2	Ref.	0.91(0.36,2.33)				
Model 3	Ref.	0.51(0.18,1.55)				

Vitamin	Group		Quartile-based Group			
	Normal	Deficiency	Q_1	Q_2	Q_3	Q_4
CHD						
Model 1	Ref.	1.63(0.68,3.93				
Model 2	Ref.	1.29(0.52,3.19)				
Model 3	Ref.	1.43(0.57,3.60)				
HTN						
Model 1	Ref.	1.31(0.97,1.77)				
Model 2	Ref.	1.23(0.89,1.69)				
Model 3	Ref.	1.27(0.91,1.78)				
DYS						
Model 1	Ref.	0.92(0.66,1.30)				
Model 2	Ref.	1.13(0.79,1.63)				
Model 3	Ref.	1.17(0.81,1.68)				
DM						
Model 1	Ref.	0.77(0.48,1.23)				
Model 2	Ref.	1.14(0.70,1.88)				
Model 3	Ref.	1.13(0.67,1.92)				
**Vitamin E**						
CVD						
Model 1	Ref.	0.87(0.45,1.70)				
Model 2	Ref.	0.86(0.43,1.73)				
Model 3	Ref.	1.01(0.49,2.09)				
CHD						
Model 1	Ref.	1.74(0.89,3.39)				
Model 2	Ref.	1.96(0.94,4.08)				
Model 3	Ref.	2.30(0.97,4.92)				
HTN						
Model 1	Ref.	1.04(0.83,1.31)				
Model 2	Ref.	1.09(0.86,1.38)				
Model 3	Ref.	1.11(0.87,1.43)				
DYS						

Vitamin	Group		Quartile-based Group			
	Normal	Deficiency	Q_1	Q_2	Q_3	Q_4
Model 1	Ref.	1.25(0.96,1.64)				
Model 2	Ref.	0.76(0.58,1.00)				
Model 3	Ref.	0.73(0.55,0.97)				
DM						
Model 1	Ref.	1.09(0.74,1.60)				
Model 2	Ref.	1.09(0.73,1.63)				
Model 3	Ref.	1.16(0.76,1.77)				
**Vitamin B1**						
CVD						
Model 1			Ref.	1.85(0.68,5.02)	**2.72(1.06,7.00)***	**3.99(1.61,9.85)****
Model 2			Ref.	2.10(0.71,6.20)	**3.17(1.14,8.86)***	**6.07(2.12,17.42)*****
Model 3			Ref.	1.94(0.64,5.89)	**2.97(1.03,8.56)***	**6.38(2.17,18.81)*****
CHD						
Model 1			Ref.	**9.07(1.14,71.78)***	**15.37(2.02,116.72)****	**15.47(2.04,117.45)****
Model 2			Ref.	6.89(0.86,55.21)	6.58(0.82,52.77)	4.84(0.57,41.10)
Model 3			Ref.	6.18(0.75,50.73)	6.79(0.83,55.39)	4.46(0.51,38.96)
HTN						
Model 1			Ref.	1.01(0.76,1.34)	**1.57(1.20,2.05)*****	**1.36(1.04,1.79)***
Model 2			Ref.	0.99(0.74,1.32)	**1.27(1.01,1.70)***	1.11(0.79,1.57)
Model 3			Ref.	0.98(0.73,1.34)	**1.36(1.01,1.85)***	1.14(0.79,1.64)
DYS						
Model 1			Ref.	1.09(0.81,1.49)	1.31(0.97,1.77)	1.26(0.93,1.70)
Model 2			Ref.	1.14(0.83,1.55)	1.36(0.99,1.87)	1.51(1.04,2.19)
Model 3			Ref.	1.13(0.82,1.55)	1.37(0.99,1.89)	1.55(1.06,2.26)
DM						
Model 1			Ref.	1.53(0.91,2.59)	**1.86(1.11,3.10)***	**3.02(1.87,4.87)*****
Model 2			Ref.	1.39(0.81,2.40)	1.15(0.65,2.01)	1.59(0.88,2.89)
Model 3			Ref.	1.45(0.82,2.58)	1.19(0.67,2.15)	1.65(0.87,3.12)
**Vitamin B2**						
CVD						

Vitamin	Group		Quartile-based Group			
	Normal	Deficiency	Q_1	Q_2	Q_3	Q_4
Model 1			Ref.	0.45(0.16,1.31)	1.85(0.88,3.88)	1.87(0.89,3.94)
Model 2			Ref.	0.51(0.17,1.49)	1.92(0.86,4.27)	2.14(0.93,4.92)
Model 3			Ref.	0.55(0.18,1.72)	1.38(0.55,3.45)	1.02(0.38,2.73)
CHD						
Model 1			Ref.	1.62(0.53,4.97)	**3.25(1.19,8.94)***	2.25(0.78,6.52)
Model 2			Ref.	1.43(0.46,4.47)	1.34(0.46,3.96)	0.57(0.17,1.84)
Model 3			Ref.	1.72(0.50,5.92)	1.53(0.46,5.12)	0.69(0.19,2.54)
HTN						
Model 1			Ref.	0.87(0.86,0.65,1.14)	1.11(0.85,1.46)	**1.40(1.07,1.82)***
Model 2			Ref.	0.84(0.63,1.12)	0.95(0.71,1.27)	1.10(0.80,1.50)
Model 3			Ref.	0.93(0.69,1.25)	0.98(0.72,1.33)	1.19(0.86,1.67)
DYS						
Model 1			Ref.	0.91(0.69.1.22)	0.80(0.60,1.08)	0.75(0.56,1.01)
Model 2			Ref.	0.88(0.66,1.18)	0.73(0.53,1.00)	0.69(0.48,0.98)
Model 3			Ref.	0.93(0.69,1.24)	0.77(0.56,1.06)	0.72(0.51,1.04)
DM						
Model 1			Ref.	1.30(0.77,2.20)	**1.89(1.15,3.08)***	**2.65(1.66,4.24)*****
Model 2			Ref.	1.21(0.71,2.05)	1.07(0.63,1.83)	1.25(0.72,2.16)
Model 3			Ref.	1.22(0.70,2.13)	0.99(0.56,1.74)	1.26(0.71,2.24)
**Vitamin B3**						
CVD						
Model 1			Ref.	1.08(0.50,2.32)	1.07(0.50,2.29)	1.16(0.55,2.46)
Model 2			Ref.	1.04(0.47,2.31)	1.13(0.52,2.47)	1.11(0.51,2.43)
Model 3			Ref.	0.74(0.31,1.76)	1.07(0.47,2.43)	1.00(0.44,2.29)
CHD						
Model 1			Ref.	1.59(0.61,4.12)	1.14(0.41,3.15)	2.03(0.81,5.07)
Model 2			Ref.	1.46(0.53,4.02)	0.93(0.31,2.82)	2.01(0.76,5.34)
Model 3			Ref.	1.26(0.45,3.54)	0.51(0.14,1.84)	1.84(0.69,4.93)
HTN						
Model 1			Ref.	1.15(0.88,1.51)	1.12(0.85,1.47)	1.22(0.93,1.59)

Vitamin	Group		Quartile-based Group			
	Normal	Deficiency	Q_1	Q_2	Q_3	Q_4
Model 2			Ref.	1.05(0.80,1.40)	1.02(0.76,1.35)	1.15(0.87,1.52)
Model 3			Ref.	1.06(0.79,1.42)	1.03(0.76,1.39)	1.14(0.85,1.53)
DYS						
Model 1			Ref.	0.90(0.67,1.22)	0.90(0.66,1.21)	1.19(0.89,1.59)
Model 2			Ref.	0.87(0.64,1.19)	0.85(0.62,1.16)	1.19(0.89,1.61)
Model 3			Ref.	0.88(0.64,1.21)	0.84(0.61,1.15)	1.17(0.87,1.59)
DM						
Model 1			Ref.	0.96(0.63,1.48)	0.91(0.59,1.41)	0.83(0.53,1.29)
Model 2			Ref.	0.81(0.52,1.27)	0.76(0.48,1.20)	0.72(0.46,1.15)
Model 3			Ref.	0.76(0.47,1.22)	0.78(0.48,1.26)	0.70(0.43,1.14)
**Vitamin B5**						
CVD						
Model 1			Ref.	1.38(0.55,3.45)	2.29(0.99,5.29)	**2.42(1.05,5.57)***
Model 2			Ref.	1.87(0.69,5.08)	**3.09(1.21,7.89)***	**3.00(1.15,7.83)***
Model 3			Ref.	1.94(0.71,5.31)	2.59(0.99,6.77)	**2.83(1.07,7.52)***
CHD						
Model 1			Ref.	0.57(0.17,1.96)	1.58(0.61,4.10)	**2.62(1.09,6.30)***
Model 2			Ref.	0.46(0.13,1.61)	0.84(0.31,2.30)	0.82(0.30,2.22)
Model 3			Ref.	0.22(0.05,1.09)	0.73(0.31,2.37)	0.86(0.31,2.37)
HTN						
Model 1			Ref.	1.18(0.88,1.58)	**1.56(1.18,2.07)****	**2.06(1.57,2.71)*****
Model 2			Ref.	1.11(0.82,1.50)	**1.37(1.02,1.85)***	**1.63(1.20,2.22)****
Model 3			Ref.	1.11(0.81,1.52)	1.33(0.98,1.82)	**1.65(1.19,2.28)****
DYS						
Model 1			Ref.	**1.55(1.12,2.14)****	**1.64(1.19,2.26)****	**2.01(1.47,2.74)*****
Model 2			Ref.	**1.48(1.06,2.06)***	**1.57(1.13,2.19)****	**1.48(1.06,2.06)*****
Model 3			Ref.	**1.48(1.06,2.08)***	**1.63(1.17,2.29)****	**2.15(1.51,3.06)*****
DM						
Model 1			Ref.	0.68(0.38,1.22)	1.59(0.99,2.57)	**2.84(1.83,4.43)*****
Model 2			Ref.	0.64(0.36,1.17)	1.19(0.71,1.99)	1.58(0.94,2.64)

Vitamin	Group		Quartile-based Group			
	Normal	Deficiency	Q_1	Q_2	Q_3	Q_4
Model 3			Ref.	0.63(0.34,1.18)	1.09(0.64,1.87)	1.57(0.92,2.69)
**Vitamin B6**						
CVD						
Model 1			Ref.	**0.44 (0.22,0.87)***	**0.32(0.15,0.70)****	**0.29(0.13,0.63)****
Model 2			Ref.	**0.42(0.20,0.87)***	**0.34(0.15,0.76)****	**0.26(0.11,0.63)****
Model 3			Ref.	**0.34(0.15,0.75)****	**0.35(0.16,0.80)***	**0.21(0.82,0.56)****
CHD						
Model 1			Ref.	0.47(0.21,1.05)	**0.36(0.15,0.87)***	**0.26(0.10,0.69)****
Model 2			Ref.	0.85(0.37,1.93)	1.33(0.48,3.69)	0.72(0.25,2.10)
Model 3			Ref.	0.80(0.33,1.91)	1.34(0.47,3.84)	0.41(0.11,1.53)
HTN						
Model 1			Ref.	0.83(0.634,1.07)	**0.63(0.48,0.83)*****	0.82(0.63,1.06)
Model 2			Ref.	1.04(0.77,1.37)	0.87(0.63,1.19)	1.05(0.79,1.43)
Model 3			Ref.	1.01(0.75,1.36)	0.85(0.61,1.18)	1.00(0.73,1.38)
DYS						
Model 1			Ref.	1.02(0.76,1.37)	0.91(0.67,1.23)	1.16(0.86,1.55)
Model 2			Ref.	1.07(0.78,1.48)	0.92(0.65,1.29)	1.11(0.80,1.54)
Model 3			Ref.	1.06(0.77,1.47)	0.89(0.63,1.26)	1.06(0.76,1.48)
DM						
Model 1			Ref.	0.71(0.48,1.05)	**0.38(0.24,0.61)*****	**0.41(0.26,0.64)*****
Model 2			Ref.	1.03(0.68,1.57)	0.77(0.45,1.33)	0.73(0.44,1.22)
Model 3			Ref.	0.99(0.63,1.54)	0.82(0.47,1.45)	0.77(0.45,1.32)
**Vitamin B9**						
CVD						
Model 1			Ref.	0.72(0.36,1.45)	0.57(0.27,1.20)	0.62(0.30,1.29)
Model 2			Ref.	0.81(0.39,1.65)	0.64(0.30,1.38)	0.65(0.30,1.40)
Model 3			Ref.	1.04(0.48,2.26)	0.82(0.36,1.85)	0.97(0.43,2.21)
CHD						
Model 1			Ref.	1.40(0.62,3.17)	0.69(0.26,1.83)	0.90(0.36,2.22)
Model 2			Ref.	1.70(0.73,3.92)	0.81(0.30,2.17)	0.95(0.35,2.57)

Vitamin	Group		Quartile-based Group			
	Normal	Deficiency	Q_1	Q_2	Q_3	Q_4
Model 3			Ref.	1.38(0.56,3.39)	0.77(0.28,2.09)	0.90(0.33,2.47)
HTN						
Model 1			Ref.	0.97(0.75,1.27)	0.85(0.65,1.10)	0.84(0.64,1.09)
Model 2			Ref.	1.03(0.78,1.36)	0.85(0.64,1.12)	0.88(0.67,1.17)
Model 3			Ref.	0.96(0.71,1.27)	0.80(0.60,1.08)	0.84(0.63,1.13)
DYS						
Model 1			Ref.	0.95(0.71,1.28)	0.97(0.73,1.30)	0.87(0.65,1.17)
Model 2			Ref.	0.99(0.73,1.34)	0.95(0.70,1.28)	0.87(0.64,1.19)
Model 3			Ref.	0.94(0.69,1.28)	0.92(0.68,1.26)	0.87(0.64,1.19)
DM						
Model 1			Ref.	0.79(0.52,1.20)	0.72(0.47,1.11)	0.69(0.45,1.07)
Model 2			Ref.	0.80(0.51,1.25)	0.71(0.45,1.12)	0.82(0.52,1.29)
Model 3			Ref.	0.66(0.41,1.05)	0.54(0.33,0.89)	0.71(0.45,1.14)

Model 1 was a crude model. Model 2 was adjusted for sex, age and education status.

CVD, CHD and HTN: Model 3 additionally adjusted BRI, smoking, alcohol consumption, exercise, and diseases that correspond to the history of illness, as indicated by homocysteine levels.

DYS: Model 3 additionally adjusted BRI, smoking, alcohol consumption, exercise, and diseases that correspond to the history of illness, as indicated by triglycerides, total cholesterol and low-density lipoprotein cholesterol (LDL-C).

T2DM: Model 3 additionally adjusted BRI, smoking, alcohol consumption, exercise, and diseases that correspond to the history of illness, as indicated by fasting blood glucose, homocysteine levels and glycosylated hemoglobin.

Blood concentrations of B vitamins were categorized into quartiles, specifically Q1 (representing the bottom 25%), Q2 (ranging from 25% to 50%), Q3 (spanning from 50% to 75%), and Q4 (encompassing the top 25%).

All of the above covariate types were grouped variables, except for the routine blood indicators, for which detailed information on grouping has been provided in the original article.

*$P < 0.05$, **$P < 0.01$, ***$P < 0.001$

CVD: Cerebrovascular Disease

CHD: Coronary Heart Disease

HTN: Hypertension

DYS: Dyslipidemia

T2DM: Type-2 Diabetes Mellitus

6 Additional file S6. Age as an interaction term to analyze the association between vitamins and age-related diseases

6.1 Supplemental Table S8. Age as an Interaction Factor to Explore Logit Models for Vitamins and Age-Related Diseases

Interaction: Age	β	P-value
**Vitamin A (Ref:Normal)**		
HTN	-0.0058	**0.036**
DYS	-0.0114	**<0.001**
T2DM	-0.0139	**0.004**
**Vitamin B_1 (Ref:Q_1)**		
CVD		
Q_2	0.0069	0.494
Q_3	0.0018	0.861
Q_4	0.0016	0.868
HTN		
Q_2	-0.0027	0.375
Q_3	0.0006	0.825
Q_4	-0.0065	**0.003**
**Vitamin B_5 (Ref:Q_1)**		
CVD		
Q_2	0.0001	0.996
Q_3	0.0019	0.796
Q_4	-0.0081	0.296
HTN		
Q_2	0.0001	0.765
Q_3	0.0029	0.314
Q_4	0.0024	0.405
DYS		
Q_2	0.0088	**0.01**
Q_3	0.0095	**0.005**
Q_4	0.0125	**<0.001**
**Vitamin B_6 (Ref:Q_1)**		
CVD		
Q_2	0.0026	0.649
Q_3	0.0074	0.714
Q_4	-0.0007	0.992

Logit model was adjusted for sex, age, education status, BRI, smoking, alcohol consumption, exercise, and diseases that correspond to the history of illness, as indicated by homocysteine levels.

CVD, CHD and HTN: Homocysteine levels.

DYS: Triglycerides, total cholesterol and low-density lipoprotein cholesterol (LDL-C).

T2DM: Fasting blood glucose, homocysteine levels and glycosylated hemoglobin.

Blood concentrations of B vitamins were categorized into quartiles, specifically Q1 (representing the bottom 25%), Q2 (ranging from 25% to 50%), Q3 (spanning from 50% to 75%), and Q4 (encompassing the top 25%).

CVD: Cerebrovascular Disease

CHD: Coronary Heart Disease

HTN: Hypertension

DYS: Dyslipidemia

T2DM: Type-2 Diabetes Mellitus

7 Additional file S7. Results of sensitivity analysis

7.1 Supplemental Figure S2. Sensitivity Analysis of Restricted Cubic Splines

7.1.1 A Equidistant knots (P30,P60,P90)

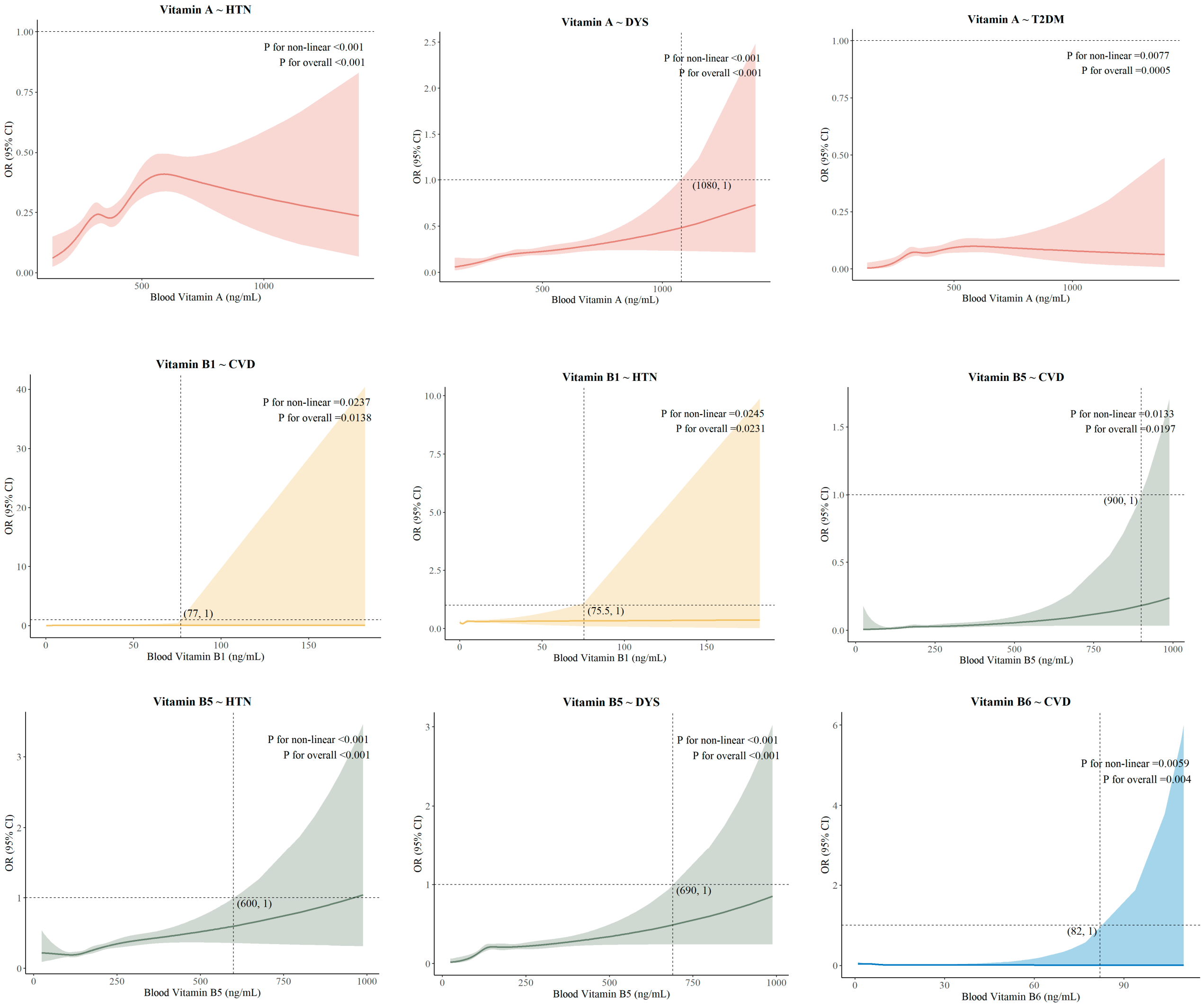

7.1.2 B Four knots (P_{20}, P_{40}, P_{60}, P_{80})

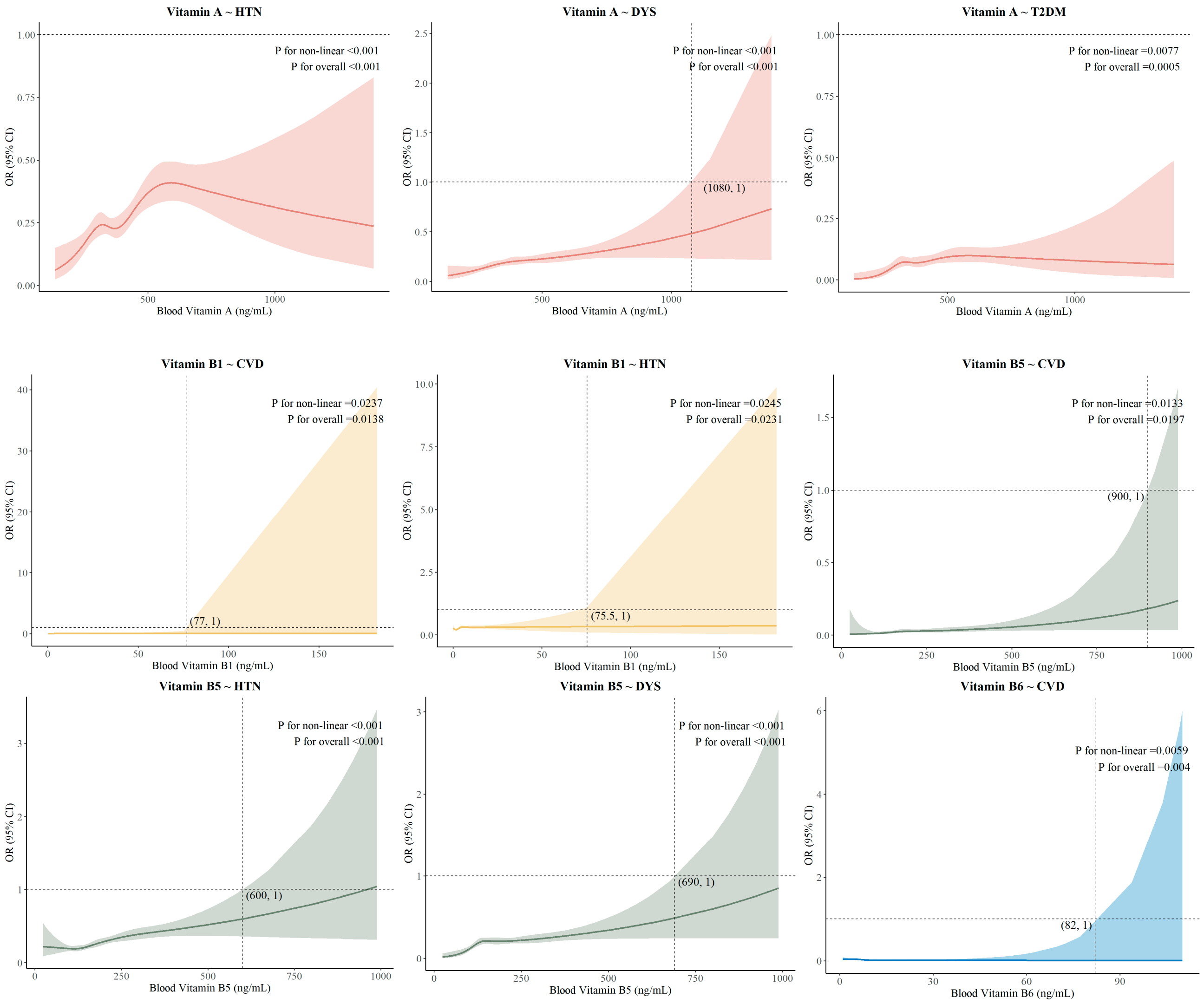

7.2 Supplemental Figure S3. Performance evaluation (ROC curve) of the fully adjusted logistic model used for the RCS method

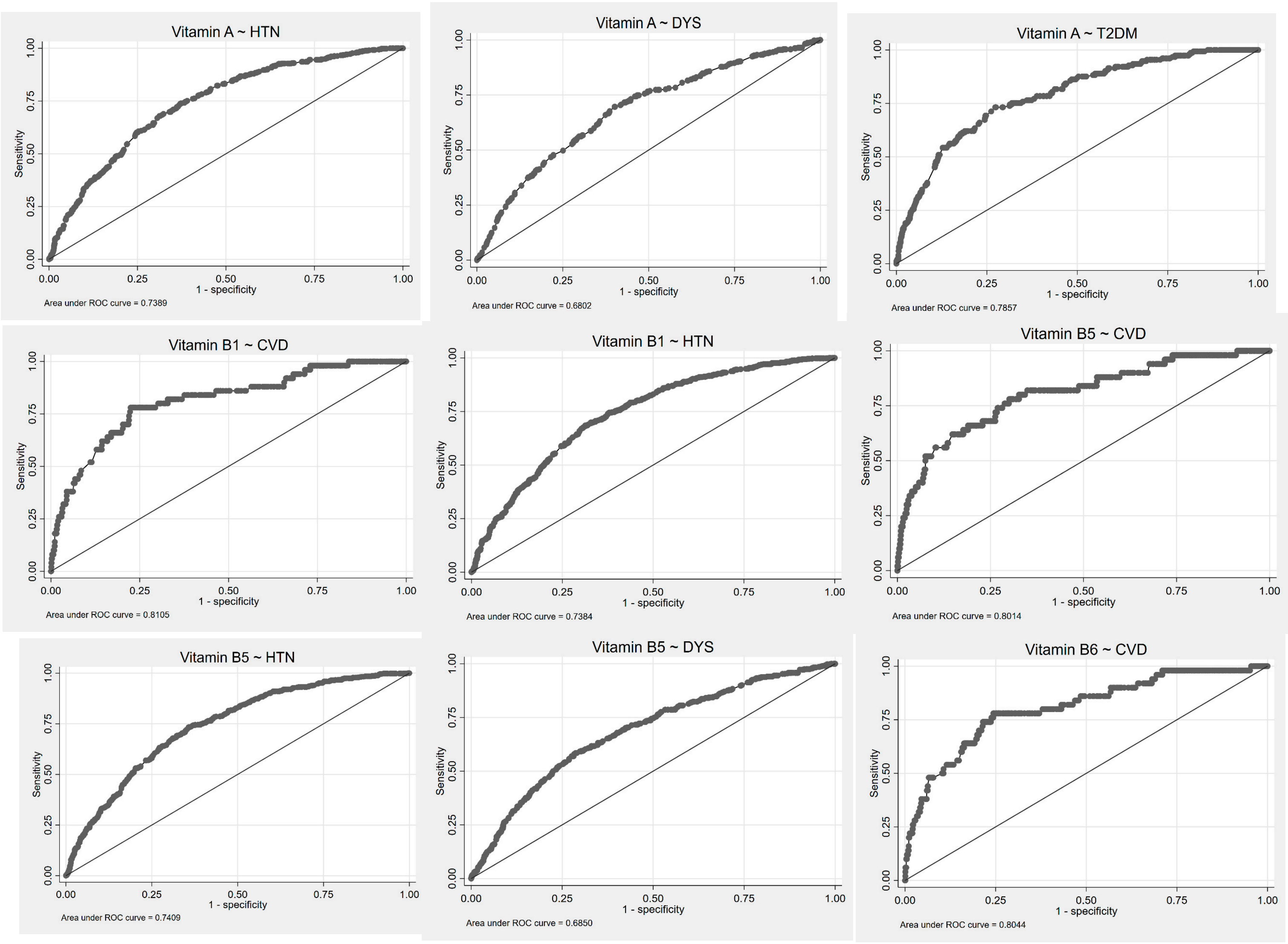

Supplement: Supplementary file 1 [file nutrients-17-01637-s001.zip › nutrients-3626483-supplementary.pdf]
